# Supplementary material for: Evolution of Anti-RBD IgG Avidity following SARS-CoV-2 Infection
Source: Viruses. 2022 Mar 4;14(3):532. doi: 10.3390/v14030532 (PMC8949389; doi:10.3390/v14030532)
Supplement: Supplementary file 1 [file viruses-14-00532-s001.zip › viruses-1614783-supplementary.pdf]

Supplementary Materials

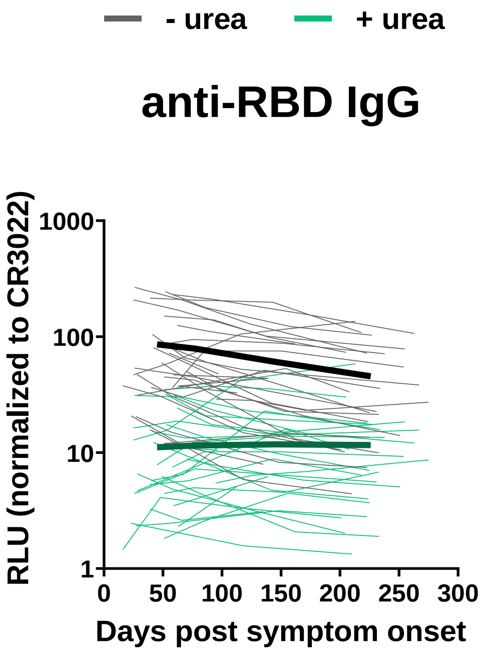

Figure S1. Comparison of the detection of RBD specific antibodies between ELISA and stringent ELISA. Level of RBD-Specific IgG was measured by indirect ELISA and stringent ELISA. Anti-RBD Ab binding was detected using HRP-conjugated anti-human IgG. Relative light unit (RLU) values obtained were normalized to the signal obtained with the anti-RBD CR3022 mAb present in each plate. For ELISA (black curves), all the wash steps were made with washing buffer and for stringent ELISA (green curves), the wash steps were made with 8M of urea. Each curve represents the normalized RLUs obtained with the plasma of one donor at every time point. Mean of each group is represented by a bold line.

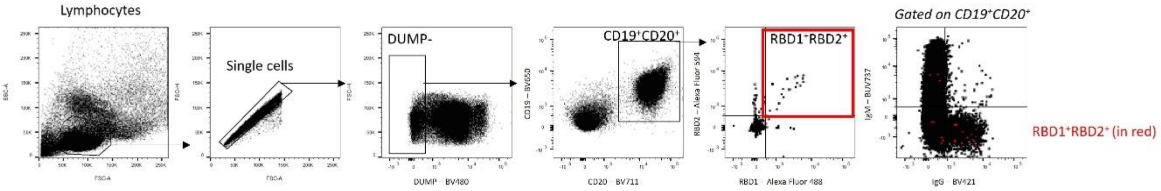

Figure S2. Gating strategy for SARS-CoV-2-specific B cell characterization. Representative flow cytometry gates to identify RBD-specific B cells from PBMCs of one convalescent donor 6 weeks PSO..
